# The Economic Impacts of Myalgic Encephalomyelitis/Chronic Fatigue Syndrome in an Australian Cohort

**DOI:** 10.3389/fpubh.2020.00420

**Published:** 2020-08-21

**Authors:** Shara Close, Sonya Marshall-Gradisnik, Joshua Byrnes, Peter Smith, Son Nghiem, Don Staines

**Affiliations:** ^1^Menzies School of Health Research, Charles Darwin University, Darwin, NT, Australia; ^2^National Centre for Neuroimmunology and Emerging Diseases, Griffith University, Gold Coast, QLD, Australia; ^3^Centre for Applied Health Economics, Griffith University, Nathan, QLD, Australia

**Keywords:** myalgic encephalomyelitis, chronic fatigue syndrome, health economics, public health, economic impact, out of pocket cost, health care service utilization, diagnostics

## Abstract

**Objectives:** This study aims to estimate direct and indirect health economic costs associated with government and out-of-pocket (OOP) expenditure based on health care service utilization and lost income of participants and carers, as reported by Australian Myalgic Encephalomyelitis/Chronic Fatigue Syndrome (ME/CFS) patient survey participants.

**Design:** A cost of illness study was conducted to estimate Australian cost data for individuals with a ME/CFS diagnosis as determined by the Canadian Consensus Criteria (CCC), International Consensus Criteria (ICC), and the 1994 CDC Criteria (Fukuda).

**Setting and participants:** Survey participants identified from a research registry database provided self-report of expenditure associated with ME/CFS related healthcare across a 1-month timeframe between 2017 and 2019.

**Main outcome measures:** ME/CFS related direct annual government health care costs, OOP health expenditure costs, indirect costs associated with lost income and health care service use patterns.

**Results:** The mean annual cost of health care related expenditure and associated income loss among survey participants meeting diagnostic criteria for ME/CFS was estimated at $14.5 billion. For direct OOP and Government health care expenditure, high average costs were related to medical practitioner attendance, diagnostics, natural medicines, and device expenditure, with an average attendance of 10.6 referred attendances per annum and 12.1 GP visits per annum related specifically to managing ME/CFS.

**Conclusions:** The economic impacts of ME/CFS in Australia are significant. Improved understanding of the illness pathology, diagnosis, and management, may reduce costs, improve patient prognosis and decrease the burden of ME/CFS in Australia.

## Introduction

Myalgic Encephalomyelitis/Chronic Fatigue Syndrome (ME/CFS), is a debilitating, chronic illness with a high level of social and economic burden due to its disabling, widespread chronic pain, and negative impacts on cognition and multiple body systems (1). The complexity of ME/CFS is compounded by its heterogeneity across onset, symptomatology, relapsing nature, and varying levels of severity ranging from mild impairment to bedridden. ME/CFS often incapacitates individuals over a long period of time, often with a prognosis of increasing severity.

The pathomechanism of ME/CFS is not well-defined. There is no specific laboratory-based diagnostic test and 20 different case definitions have been published (2). Diagnosis relies on assessing patient-reported symptoms to establish whether patients meet a specified case definition, along with extensive testing to exclude other illnesses or causative factors. The use of varying case definition criteria which range from overly broad to highly specified increases the likelihood that persons without ME/CFS are included in clinical trials and interventions distorting clinical trial outcomes and increasing costs for the health care system (1). Understanding the pathology of illness is critical to undertaking robust clinical trials to develop and test diagnostics, treatments, therapeutics and effective clinical management. In the absence of a defined pathology and diagnostic test, it is difficult to establish effective strategies to mitigate the burden and cost of illness.

This study uses three diagnostic criteria; 1994 CDC (Fukuda), Canadian Consensus Criteria (CCC) and International Consensus Criteria (ICC), as outlined in Table 1, to ascertain whether participants meet a case definition for ME/CFS. In a recent ME/CFS advisory report, the ICC and the CCC were identified as the most appropriate case definitions and are recommended for future use. The Fukuda definition, while criticized for being overly broad (3) and potentially resulting in false-positive diagnoses, was included as a diagnostic criterion in this study to allow for comparison with international ME/CFS cost of illness studies (4) and to quantify the costs of participants meeting the Fukuda diagnostic criteria for ME/CFS, as these may be indicative of the costs associated with a misdiagnosis of ME/CFS.

**Table 1:** Case definitions for ME/CFS.

**Fukuda (1994 CDC)**	**2011 International Consensus**	**Canadian Consensus**
**REQUIRED PRIMARY SYMPTOM/S**		
**Chronic debilitating fatigue** present for longer than 6 months not relieved by rest, and not due to ongoing exertion	**Post-exertional fatigue** Prolonged, persistent or relapsing, that has been present for longer than 6 months not relieved by rest, and not due to ongoing exertion	**Fatigue, post-exertional malaise and/or fatigue, sleep dysfunction and pain** that persists for at least 6 months
**REQUIRED ACCOMPANYING SYMPTOMS**		
(4 of the following) Post-exertional malaise Impaired memory or concentration Headaches Muscle pain Joint pain Unrefreshed sleep Sore throat Tender lymph nodes	**Neurological/cognitive**	
	(1 from each of the 4 categories) Neurocognitive Impairment -Difficulty processing information -Short term memory loss Pain/Headaches Sleep disturbance -Disturbed/Unrefreshed sleep Neurosensory, perceptual and motor disturbances: -Neurosensory and perceptual disturbances /Motor disturbances	(2 or more of the following) Confusion Impairment of concentration and short-term memory consolidationDisorientationDifficulty with information processing, categorizing and word retrieval-Perceptual and sensory disturbances
	**Immune, gastro-intestinal & genitourinary**	**Autonomic(a), Neuro endocrine(b) and Immune (c)**
	(1 of the following 5 symptom categories) -Flu-like symptoms -Susceptibility to viral infections with prolonged recovery periods -Gastro-intestinal disturbances -Genitourinary -Sensitivities	(1 of the following from 2 of the 3 categories a, b, c) **(a)** Orthostatic intolerance, neutrally mediated hypotension (NMH), postural orthostatic tachycardia syndrome (POTS), delayed postural hypotension; light-headedness; extreme pallor; nausea and irritable bowel syndrome; urinary frequency/bladder dysfunction; palpitations exertional dyspnea **(b)** Loss of thermostatic stability subnormal body temperature and marked diurnal fluctuation, sweating episodes, recurrent feelings of feverishness and cold extremities; intolerance of extremes of heat and cold; marked weight change anorexia or abnormal appetite; loss of adaptability and worsening of symptoms with stress **(c)** Tender lymph nodes, recurrent sore throat, recurrent flu-like symptoms, general malaise, new sensitivities to food, medications and/or chemicals
	**Energy production/transportation**	
	(1 of the following 4 symptom categories) -Cardiovascular -Respiratory -Loss of thermostatic stability -Intolerance to extremes of temperature	

ME/CFS patients are often undiagnosed or experience long delays until diagnosis due to the absence of a lab-based diagnostic test and a lack of General Practitioner (GP) awareness and understanding of ME/CFS (4). In addition to extensive testing, patients will often seek additional and alternative advice and therapeutic options from multiple GPs and a range of health professionals (4). Delays and ambiguity around diagnosis can result in confusion, distress, and poor illness management potentially contributing to patients developing more severe and debilitating forms of ME/CFS (5, 6). Suicide rates are reportedly higher in ME/CFS than comparable conditions (7), likely as a result of the severity and limited options to improve ME/CFS patient quality of life. Improved diagnostic timeframes and clinical management are critical for improving patient outcomes and reducing the burden and economic impact of ME/CFS (4).

No cost-of-illness studies have recently been undertaken in an Australian ME/CFS cohort. Understanding the financial burden associated with ME/CFS and better recognition of the condition will reduce negative impacts on patients and the health care system, progress equitable care for people with ME/CFS, and potentially mitigate high expenditure (1, 8, 9). This study aims to identify epidemiological factors, patient behaviors and expenditure associated with ME/CFS in Australia to improve an understanding of the economic impacts and better inform strategies to mitigate the burden and costs of ME/CFS.

## Materials and Methods

A cross-sectional economic survey was used to capture indirect and direct costs associated with ME/CFS across 2017–2019. All resource use attributed to ME/CFS was derived using a self-completed online survey. Participants were recruited from the Australian ME/CFS National Center for Neuroimmunology and Emerging Diseases (NCNED) Research Registry Survey database and a research participant network. The NCNED Research Registry Survey database and the research participant network include patients predominantly from across Australia with representation across multiple States and Territories, The registry has been built over the last eight 8 years to capture information about ME/CFS patients and healthy controls, who have participated in NCNED research and trials, have been targeted through ME/CFS advocacy networks or referred by medical professionals post diagnosis of ME/CFS.

Economic survey participant data were matched with participant data from the Research Registry Survey to establish demographic and illness characteristics of patients to enable criteria defined diagnosis of ME/CFS (Table 2). The economic survey captured costs directly associated with ME/CFS as identified by participants over a 1-month period immediately prior to completing the economic survey. All data were multiplied by 12 to obtain annual costs (see Table 3 below).

**Table 2:** Participant characteristics by ME/CFS definition.

**Characteristic**	**Any**	**FUKUDA**	**CCC**	**ICC**
*n* (%)^a^	85 (100%)	18 (21.2%)	23 (27.1%)	44 (51.8%)
Male (%)	24.7%	16.7%	8.7%	36.4%
Indigenous	0.0%	0.0%	0.0%	0.0%
Age (mean)	46.42	51.22	43.22	46.14
Education (%)				
High school	14.1%	16.7%	17.4%	11.4%
Postgrad	32.9%	33.3%	30.4%	34.1%
Professional	20.0%	27.8%	8.7%	22.7%
Undergrad	32.9%	22.2%	43.5%	31.8%
Employment status (%)				
Unemployed	64.7%	72.2%	56.5%	65.9%
Part time	30.6%	27.8%	39.1%	27.3%
Full time	4.7%	0.0%	4.3%	6.8%
Current income (p.a.)	$20,200	$14,281	$17,241	$24,592
Height (cm)	168.50	167.39	165.58	170.48
Weight (Kg)	77.42	84.77	72.36	77.05
BMI	27.35	30.36	26.37	26.62

a *Numbers and proportions of participants meeting the Fukuda, CCC and ICC case definitions*.

**Table 3:** Annual service utilization per person by ME/CFS definition.

	**Any**	**FUKUDA**	**CCC**	**ICC**
**Medicines**				
Prescription	16.4	16.6	17.2	15.8
Non-prescription and natural medicines	14.8	14.0	14.6	15.3
*Total medicines*	31.2	30.6	31.8	31.1
**Attendances**				
*Non*-referral				
GP	12.1	11.0	12.2	12.5
Nurse	3.4	4.9	3.2	3.0
*Total non-referral*	*15.6*	*15.8*	*15.4*	*15.5*
Referral				
Neurologist	0.6	0.3	0.6	0.8
Cardiologist	0.7	0.8	0.6	0.8
Gastro-specialist	0.5	0.3	0.6	0.5
Psychologist	4.3	3.3	4.4	4.6
Sleep-specialist	0.8	0.8	0.8	0.8
Pain-specialist	0.4	1.5	0.2	0.0
Radiologist	0.1	0.5	0.0	0.0
Other-specialist	3.1	1.5	3.0	3.8
*Total referral*	*10.6*	*8.9*	*10.2*	*11.5*
Total attendances	26.1	24.8	25.6	27.0
**Devices**	7.8	7.7	7.2	8.2
**Diagnostics**	6.5	4.9	5.0	7.9
**Hospitalizations**	2.4	2.3	2.2	2.5

The study uses a prevalence cost; an aggregate measure of the economic burden of disease reported for a specific time period. It is based on the costs of medical care (direct health system costs), costs associated with accessing care (direct patient costs) and lost income (indirect health care costs). Estimates are based on all individuals diagnosed with or living with the condition (10).

The direct health system costs include hospitalizations, prescription medication, medical devices, diagnostic tests, and attendances with medical and allied health professionals. Direct costs to patients include travel costs, OOP costs for healthcare (i.e., co-payments), non-prescription medicines and formal (i.e., paid) care and support, and insurance premiums. Indirect costs include reduced or lost income patients and carers due to ME/CFS. Cost estimates for prescription, hospitalization and medical services are based on 2019 prices.

Prevalence estimates of ME/CFS in Australia were sourced from a 2013 meta-analysis of prevalence studies (11). ME/CFS diagnostic criteria classification was undertaken using Research Registry Survey responses to ascertain whether participants met the Fukuda, ICC, CCC definitions. Classification analysis was supported by the second author of the paper, who is a specialist in ME/CFS diagnosis and along with methodologies used in other peer-reviewed studies (12). The total annual cost attributed was derived by multiplying the annual cost per person with the prevalence of ME/CFS in Australia. Cost estimates are provided by classification and for the whole ME/CFS population.

For the national cost estimate of the number of people with ME/CFS by specific classification, a hierarchical approach was applied for those who meet multiple classification definitions, based on the greater specificity and complexity of ICC and CCC case definitions. Participants meeting these case definitions are more likely to have ME/CFS. Accordingly, all respondents who met the ICC definition (regardless of also meeting other criteria) were considered as ICC and those who met the CCC and Fukuda definition were considered CCC with the remainder just meeting the Fukuda definition being classified as Fukuda. Hence these definitions are not mutually exclusive. Rather, they are overlapping with increasing levels of stringency going from Fukuda to CCC to ICC. However, to aid calculations this approach of simplified proportions was adopted. These simplified proportions of the total ME/CFS population were then applied to the total prevalence estimate of ME/CFS in Australia to estimate the prevalence of each classification.

The cost to the government was estimated based on the price of prescription medications listed on the PBS, less patient co-payment (13). Co-payments applicable to the general population (as opposed to concession card holders) were assumed. The cost of diagnostics and medical attendances was based on the MBS reimbursements (14). OOP costs were reported directly from participants. As patients with ME/CFS are unlikely to meet eligibility requirements for GP chronic disease management plans, attendances with allied health professionals were considered as OOP costs. Productivity costs were derived based on participant self-report of the difference in income pre-onset of illness and income at the time of undertaking the study.

## Results

Economic survey respondents were matched with the Research Registry Survey data. Eighty-five of the 163 respondents met one of three definition criteria for ME/CFS. Of the responders meeting a defined case definition, there were more females than males, and the mean age was approximately 46. Majority of responders had a bachelor's degree or postgraduate education (65.8%). 95.3% reported being either unemployed (64.7%) or working part-time (30.6%).

**Figure 1:**
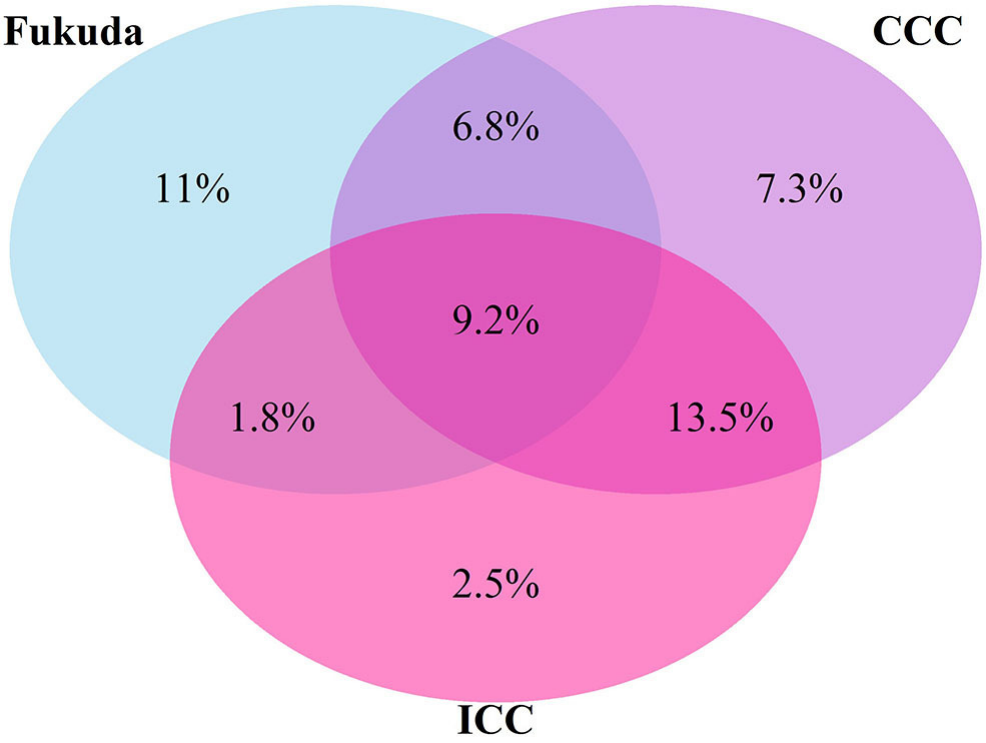
Venn diagram of proportions of participants who met one or more diagnostic Criteria for ME/CFS (%).

Seventy-eight responders (47.9%) did not meet any of the case definitions. Majority of responders met at least one of the definitions (*n* = 85), with 51.8% of those meeting the most stringent ICC definition (*n* = 44). There was overlap in the classification systems (Figure 1) with 36 participants meeting any two definitions, and 15 meeting all three definitions. Using the hierarchical approach to address the overlap between classification systems, 21.2% of participants have been classified under the Fukuda case definition and 27.1 under the CCC case definition.

Previous studies indicated that ICC defined ME/CFS represents a subgroup of ME/CFS patients with decreased physical and social functioning capacity relative to the Fukuda defined groups (6, 15). In this study, all groups reported high levels of unemployment due to illness and substantial indirect costs incurred as a result of lost income. The average annual loss in this cohort was $36,549 for Fukuda, $45,211 for ICC and $55,583 for CCC. There are higher costs and greater losses in income in the ICC and CCC cohorts compared to the Fukuda cohort.

The total average annual cost per person meeting any of the three ME/CFS definitions used in this study is $75,697. Most of the costs were borne by the patient ($71,215), compared to healthcare costs borne by the government ($4,482). Despite infrequent hospitalization (Table 4), hospital costs were the largest single cost to governments ($2,445), followed by costs for medical professional attendances ($1,123). Indirect healthcare costs (reduced patients and carers income) was the largest cost ($48,757 and $3,918, respectively) to patients, followed by direct OOP costs associated with devices, diagnostics, and medical professional attendances. The cost to individuals for natural medicines is almost two times that of prescription medication ($1,267 vs. $639).

**Table 4:** Annual average per person cost of ME/CFS based on criteria for diagnosis.

**Cost**	**Any**	**FUKUDA**	**CCC**	**ICC**
Personal Costs				
**Direct health care costs**				
Insurance premium	$1,350	$1,280	$1,294	$1,407
Attendances	$1,982	$1,530	$1,858	$2,232
Hospitals	$22	$6	$25	$27
Allied health	$1,115	$1,087	$1,193	$1,085
Diagnostics	$2,343	$1,730	$1,853	$2,848
Prescription medication	$639	$548	$682	$653
Natural Medication	$1,267	$955	$1,217	$1,421
Devices	$8,382	$1,099	$4,148	$13,561
Travel costs	$566	$822	$542	$474
Other costs	$274	$217	$556	$150
Paid support	$600	$752	$598	$540
**Total annual average direct out of pocket costs**	**$18,540**	**$10,025**	**$13,966**	**$24,398**
**Indirect health care costs**				
Reduction in Income	$48,757	$36,549	$45,211	$55,583
Reduction in carers income	$3,918	$1,128	$2,825	$5,625
**Total annual average indirect out of pocket costs**	**$52,675**	**$37,676**	**$48,036**	**$61,208**
**Total annual average Personal Cost**	$71,215	$47,701	$62,002	$85,606
Government Healthcare Costs				
***Community***				
Prescription medication	$232	$321	$206	$209
Diagnostics	$683	$488	$639	$785
Attendances	$1,123	$995	$1,110	$1,182
**Total community direct healthcare costs**	$2,037	$1,803	$1,954	$2,175
**Hospital**				
Hospitals	$2,445	$2,719	$3,288	$1,893
**Total annual average Government Healthcare Costs**	**$4,482**	**$4,523**	**$5,242**	**$4,068**
Total Combined Costs				
**Total annual average direct health care costs**^**a**^	**$23,022**	**$14,548**	**$19,208**	**$28,466**
Total annual average cost	$75,697	$52,224	$67,244	$89,674

a *Total annual average direct health care cost includes government health care costs and direct OOP costs*.

Using a national prevalence of 0.76% (11), there are an estimated 191,544 Australians living with ME/CFS. The estimated total cost of ME/CFS in Australia was $14,499 million annually (Table 5). The estimated cost to the Australian Government was $858 million per annum. Based on 95% confidence intervals of the prevalence estimates, the total cost estimate ranges from $3,335 million to $18,704 million.

**Table 5:** Estimated total cost of ME/CFS in Australia, 2017–2019.

	**Any**	**FUKUDA**	**CCC**	**ICC**
Direct OOP costs per person	$18,540	$10,025	$13,966	$24,398
Indirect costs per person	$52,675	$37,676	$48,036	$61,208
*Personal cost per person*	$71,215	$47,701	$62,002	$85,606
*Government cost per person*	$4,482	$4,523	$5,242	$4,068
Prevalance^a^	0.76%	21.1%	27.1%	51.8%
Population Estimate (N)	191,544	40,441	51,838	99,265
Direct OOP costs per person	$3,551	$405	$724	$2,422
Indirect costs per person	$10,090	$1,524	$2,490	$6,076
*Total personal cost (Mill)*	$13,641	$1,929	$3,214	$8,498
*Total government cost (Mill)*	$858	$183	272	404
*Total direct^*b*^ costs (Mill)*	**$4,409**	**$588**	**$996**	**$2,826**
**Total Cost (Mill)**	**$14,499**	**$2,112**	**3,486**	**8,901**
95% LCI	$3,335	$486	802	2,047
95% UCI	$18,704	$2,724	4,497	$11,483

a *Fukuda, CCC, ICC and no classification prevalence as a proportion of total prevalence estimate*.

b *Total direct cost includes government health care costs and direct OP costs*.

## Discussion

This study was undertaken to establish the direct and indirect economic costs associated with ME/CFS in an Australian cohort. It extrapolates those costs to the estimated Australian ME/CFS population to examine the healthcare use profile and types of expenses associated with managing ME/CFS. Of the estimated $14.5 billion annual Australian cost, 70% was due to lost income, 24% due to direct personal OOP costs on health and medical expenditure, and 6% incurred as a cost to government and the health care system.

This cost is significant and is comparable with international studies. A 2011 study in the United States (US), estimated a direct expenditure of USD$14 billion in national healthcare costs and USD$37 billion in lost productivity (16). A 2008 study estimated total direct annual costs in the order of USD$2 billion-9 billion (17) with annual direct costs up to USD$8,854 per ME/CFS patient (17). This study estimates the direct annual health care costs per patient in Australia ($23,022), including direct OOP costs per patient ($18,540) and direct government healthcare costs ($4,482), to be significantly more than the upper US estimates (see Table 4 above).

The OOP expenditure associated with ME/CFS alone in this study represents a higher proportion of OOP costs spent on health care by participants than by the overall broader Australian population, estimated to be 18% in 2009/10 (18). The average monthly direct personal cost estimated in the study was almost 10 times the estimated direct personal costs incurred (~$160 per/month) by chronic obstructive pulmonary disease, which represented 3% of the total burden of disease and injury in Australia in 2003 (19). The average direct annual OOP cost estimated in this cohort ($3.5 billion) was estimated to make up 7% of the estimated per capita OOP payments, $24.3 billion, made by Australians between 2011 and 2012 (20).

Understanding cost profiles is important to establish where high expenditure and health service use exists, and how these might influence decision making around support requirements and/or opportunities for cost reductions. This study indicated a high level of expenditure associated with natural medicines, devices and diagnostics, and a broad array of medical and allied health professional attendances. The largest OOP cost relates to devices, and the largest cost to government due to hospitalizations. Compared to the 2018 national average, patients that meet any of the ME/CFS definitions have approximately twice as many visits with a GP (12.1 vs. 6) (21).

High testing costs and medical specialist costs are associated with managing ME/CFS as there are no laboratory-based tests available to diagnose the illness and diagnosis involves testing to exclude other conditions. As the Fukuda definition has been identified as being overly broad, the costs associated with participants in this study meeting the Fukuda definition only, may be indicative of costs associated with misdiagnosis and of not having a lab-based diagnostic test. Based on the estimated prevalence cost of this study, this cost is in the order of two billion dollars.

There is presently insufficient evidence that the use of nutritional supplements and elimination or modified diets relieves ME/CFS symptoms (22). Despite this expenditure on supplements was high. In the absence of clinical evidence of effective treatments for ME/CFS (19), medications and supplements that do not alleviate symptoms of individual patients may be an unnecessary expense. Training and educating health professionals to diagnose and provide appropriate treatment may improve patient prognosis and reduce higher costs associated with more severe ME/CFS (5).

High OOP costs for long term chronic illness patients are of increasing concern to the Australian health system and society as the financial strain on individuals can result in individuals not seeking adequate health care and their condition worsening (23). This is particularly concerning for ME/CFS patients who may further avoid seeking out health care due to low expectations around receiving adequate care and support (24).

Estimated extrapolated costs in the study population are exceedingly high for a relatively small percentage of the population. Considering the low federally funded expenditure on ME/CFS research in Australia to date, there is potential to significantly reduce the public health burden of ME/CFS in Australia. Better understanding, diagnosis, treatment and management of the illness, and better support for patients and carers will be critical to reducing such costs (1, 8). A laboratory-based diagnostic test has further potential to significantly decrease costs by providing greater certainty in identifying ME/CFS patients, undertaking clinical trials, and developing appropriate treatments with proven effectiveness.

The economic impacts of ME/CFS in Australia are substantial for patients and the government. Federal ME/CFS research funding expenditure in Australia is not reflective of the significant economic impacts of the illness. The ongoing and potentially increasing financial burden will be difficult to alleviate without targeted research into the pathomechanism of ME/CFS, development of effective treatment options and improved diagnosis and management of ME/CFS patients (3). This study provides an indication that the high costs associated with ME/CFS could be significantly reduced through the development of a lab-based diagnostic, more effective treatment options and better management strategies through improved awareness and training for General and Specialist Practitioners.

## Limitations

In this study diagnosis, ME/CFS classification, attribution, and estimates of costs associated with ME/CFS were based on self-reported responses to an online survey. This method is common, despite associated known weaknesses such as recall bias. This may result in classification error, underestimate total resource consumption, and over represent significant events (such as hospitalizations) relative to minor occurrences such as medical attendances. It is unknown if this would over or underestimate the extent to which the reported healthcare use is due to ME/CFS nor what the extent of misdiagnosis might be. The sample was, however, based on participants identified through the ME/CFS research registry survey database, many of whom had received a diagnosis from a health care clinician, with validated diagnostic questions that provide a robust means for classification.

## Data Availability Statement

The raw data supporting the conclusions of this article will be made available by the authors, without undue reservation.

## Ethics Statement

The studies involving human participants were reviewed and approved by Griffith University Human Research Ethics Committee (GUHREC reference number 2016/502). The patients/participants provided their written informed consent to participate in this study.

## Author Contributions

SC, SM-G, JB, and DS contributed to conception and design of the study. SM-G and DS set up and organized the database. SC, JB, and SN performed the data and statistical analysis. SC, JB, and SN wrote the first draft of the manuscript. SC, SM-G, JB, PS, SN, and DS wrote and significantly revised sections of the manuscript. All authors contributed to manuscript revision, read, and approved the submitted version.

## Conflict of Interest

The authors declare that the research was conducted in the absence of any commercial or financial relationships that could be construed as a potential conflict of interest.
